# Les manifestations ophtalmologiques de la maladie de Behçet, à propos de 33 cas

**Published:** 2012-12-19

**Authors:** Idriss Benatiya Andaloussi, Bouchra Alami, Meryem Abdellaoui, Salima Bhallil, Wafae Bono, Hicham Tahri

**Affiliations:** 1 Service d'ophtalmologie, CHU Hassan II, Fès, Maroc; 2 Service de médecine interne, CHU Hassan II, Fès, Maroc

**Keywords:** Manifestations ophtalmologiques, maladie de Behçet, uvéite, vascularite, cécité, ophthalmologic manifestations, Behçet's disease, uveitis, vascularitis, blindness

## Abstract

Les manifestations ophtalmologiques au cours de la maladie de Behçet sont dominées par les uvéites, les vascularites rétiniennes et les thromboses veineuses rétiniennes. Nous rapportons une étude rétrospective portant sur tous les malades atteints de la maladie de Behçet, hospitalisés au sein du service d'Ophtalmologie de Fès de Janvier 2007 à Décembre 2009. Au total ce sont 33 patients qui sont inclut dans l'étudeLa moyenne d’âge est de 28,4 ans. Le délai moyen de consultation varie entre 1 jour et 3 ans. L'atteinte oculaire est bilatérale chez 26 patients (78,8% des yeux). L'AV est très basse avant le traitement: 28,8% à moins de 1/10. Les manifestations oculaires sont dominées par l'uvéite avec 77,3%, suivie de la vascularite rétinienne 54, 5% et la maculopathie 51, 5%. Le pourcentage des yeux dont l'AV était inférieur à 1/10 est passé à 19,7% après traitement. La fréquence de l'atteinte oculaire au cours de la maladie de Behçet est diversement appréciée selon les auteurs et selon le mode de recrutement des patients: elle va de 29% à 100%. L'atteinte uvéale est la plus fréquente des manifestations ophtalmologiques. Les lésions vasculaires rétiniennes sont dominées par la vascularite rétinienne essentiellement la périphlébite aussi bien au pôle postérieur qu’à la périphérie rétinienne. En l'absence de traitement et de mauvaise observance, le pronostic oculaire de la maladie de Behçet est très mauvais. La cécité s'installe dans 13 à 32%. Les manifestations ophtalmologiques au cours de la maladie de Behçet ont une valeur considérable, tant sur le plan diagnostique que pronostique. Notre étude a permis de décrire les aspects cliniques de l'atteinte oculaire de la maladie de Behçet dans un service d'Ophtalmologie tertiaire au Maroc.

## Introduction

La maladie de Behçet est une maladie inflammatoire chronique, évoluant par poussées entrecoupées de rémission, caractérisée cliniquement par: des ulcérations orales et génitales; des lésions cutanées (pseudofolliculite nécrotique, érythème noueux); des manifestations systémiques: oculaires (uvéite, vascularite rétinienne), neurologiques (encéphalomyélite, méningite, hypertension intracrânienne), et vasculaires (thromboses, anévrysmes).

Le substratum anatomique commun à ces différentes atteintes est une vascularite capable de toucher tous les vaisseaux, quels que soient leur nature et leur calibre, avec néanmoins une prédominance pour l′atteinte veineuse [1, 2]. Les manifestations ophtalmologiques au cours de la maladie de Behçet sont dominées par les uvéites, les vascularites rétiniennes et les thromboses veineuses rétiniennes. L′atteinte du nerf optique est moins fréquente et s'intègre parmi les manifestations neuro-ophtalmologiques de cette affection.

## Méthodes

Notre étude a porté sur l'analyse rétrospective de tous les malades atteints de la maladie de Behçet, hospitalisés au service d′Ophtalmologie du CHU Hassan II de Fès de Janvier 2007 à Décembre 2009, pour une atteinte oculaire. Seuls les patients dont les critères diagnostiques de l'International Study Group (ISG) [3] étaient complets sont retenus. Les patients atteints de maladie de Behçet, pris en charge dans d′autres unités (dermatologie, rhumatologie, neurologie, médecine interne,...) ont été exclus de l′étude.

Les renseignements ont été recueillis grâce à une fiche d'exploitation préalable précisant les paramètres suivants: âge au moment du diagnostic de la maladie, sexe, date d'apparition de l'atteinte oculaire par rapport à la date du diagnostic de la maladie de Behçet, les signes ophtalmologiques motivant la consultation, ainsi que le délai de consultation, et les données de l'examen ophtalmologique initial (mesure de l'acuité visuelle, examen à lampe à fente, fond doeil). Les manifestations extra-ophtalmologiques de la maladie ont été également notées. Le pathergy test était réalisé lorsqu'il n'y avait pas assez de critères pour retenir la maladie ou si la maladie de Behçet n’était pas connue. Les données de l'angiographie à la fluorescéine, de l'échographie oculaire, de l'OCT, du champ visuel et du test des couleurs ont été notées lorsqu'ils ont été réalisés.

Les patients présentant une uvéite ont bénéficié d'un bilan infectieux complet à la recherche de foyers infectieux potentiels pouvant expliquer les rechutes. Les différentes thérapeutiques instaurées ont été notés ainsi que le profil évolutif de l'atteinte ophtalmologique. L'existence de complications oculaires ou celles induites par les traitements a été notée. Le nombre de poussées, les causes de récidive et de rechutes ont été également rapporté. Les résultats sont exprimés en nombre d'yeux dans les différentes atteintes oculaires.

## Résultats

Au total ce sont 33 patients qui sont inclut dans l'étude, répartis en 23 hommes et 10 femmes, soit un sex-ratio H/F de 2,3. La moyenne d’âge dans notre série au moment de l'hospitalisation est de 28,4 ans (ET= 7,96 / 14 à 42 ans). La tranche d’âge la plus touchée est celle comprise entre 20 et 35 ans qui représente 54,5%. l'âge moyen au début des signes est de 26 ans ± 8.

La forme juvénile de la maladie de Behçet (< 16 ans) est retrouvée dans 4 cas, soit 12,1%. Les antécédents familiaux de maladie de Behçet sont retrouvés chez deux malades, soit 6% de l'ensemble des patients. Le délai moyen de consultation varie entre 1 jour et 3 ans, avec une moyenne de 135 jours. La majorité des patients consultent dans un délai inférieur à 1 mois (48,5%). Toutefois, 27,3% des patients consultent dans un délai entre 1mois et 6 mois, et 18,2% dans un délai de plus de 6 mois

Les manifestations extra oculaires retrouvées chez nos patients sont illustrés dans le Tableau 1. Le motif de consultation principal est la baisse de l'acuité visuelle rencontrée chez 29 patients, suivie de la rougeur oculaire, qui est retrouvée chez 21 patients, la douleur péri-oculaire dans 16 cas et les myodesopsies chez 11 patients.

**Tableau 1:** Fréquence des manifestations extra-ophtalmologique en fonction du sexe dans notre série

Signe extra ophtalmologique.	Pourcentage (%) : N= 33 patients
Aphtose buccale	100
Aphtose génitale	78,8
Pseudofolliculite	48,5
Erythème noueux	9,1
Hypersensibilité cutanée	39,1
Manifestations vasculaires	6,1
Manifestations articulaires	27,3
Manifestations neurologiques	12,1

L'atteinte oculaire est inaugurale de la maladie de Behçet dans 7 cas (21%). L'atteint oculaire est survenue 32 mois en moyenne (soit deux ans et 8 mois) après l'apparition des signes cutanées (extrêmes: 0 -10 ans). Le mode d'installation des manifestations ophtalmologiques est le plus souvent progressif (70%). L'atteinte oculaire est bilatérale chez 26 patients, et unilatérale dans 7 cas, ce qui correspond à 59 yeux atteints. La majorité des malades avaient une AV très basse avant le traitement, 28,8% CLD, 18,2% avaient une AV entre 1/10 et 3/10.

Les aspects cliniques du Behçet oculaire dans notre série, notés sur la base de l'examen ophtalmologique et des données paracliniques (angiographie, OCT), sont dominés par l'uvéite avec 77,3% des yeux atteints suivie par la vascularite rétinienne (54,2%), puis la maculopathie (50,8%) et la neuropathie optique: 47,4%. Il faut noter que ces atteintes sont associées les unes aux autres dans la plupart des cas (Tableau 2).

**Tableau 2:** Fréquence des complications ophtalmologiques chez nos patients

Complications ophtalmologiques	Nombre n= 59 yeux	Pourcentage%
Cataracte	18	30,5
Synéchie iridocristalliniennes	11	18,6
Secclusion pupillaire	6	10,1
Hypertonie oculaire	10	16,9
Glaucome	8	13,5
Rubéose irienne	1	1,7
Ischémie rétinienne	5	8,4
Trou maculaire	4	6,7
Dégénérescence maculaire	19	32,2
Pâleur papillaire	4	6,7
Néovaisseaux	3	5
Hémorragie de vitré	2	3,3
Décollement de rétine	8	13,5
Cécité	13	22

Le choix du traitement dépendait du type de l'atteinte oculaire et de sa sévérité. La corticothérapie en collyre était prescrite en association à des mydriatiques chez 97% des patients. En outre, les injections péri-oculaires de Célestène^®^ étaient préconisées chez 14 patients (42,4%) pour juguler une inflammation sévère du segment postérieur, compliquée ou non doedème maculaire. La voie latéro-bulbaire était utilisée dans 13 cas. Le bolus de Méthylprednisolone à raison de 10 mg/Kg/jour en perfusion de 3 heures pendant 3 jours a été administré à 31 patients (94%). Le relais par de la prednisone par voie orale à raison de 1 à 2 mg/Kg/jour a été assuré chez ces patients. Ce traitement était indiqué dans les atteintes du segment postérieur. Les immunosuppresseurs étaient prescrits en cas d'atteinte postérieure ou de vascularite chez 24 patients (72,7%). Le Cyclophosphamide était administré sous forme de bolus de 400mg/m2 à J1-J15-J30-J60-J90-J120 avec des cures trimestrielles en l'absence de relais par l'Azathioprine dans 17 cas. L'azathioprine (Imurel^®^) a été prescrit en relais aux cures d'Endoxan^®^ dans 5 cas, et seul chez une patiente de 21 ans.

Les poussées favorisées par la corticodépendance étaient jugulées par une augmentation des doses, alors que celles favorisées par les causes infectieuses ont justifié l'usage des antibiotiques en fonction du germe incriminé.

Une nette amélioration de l'AV est observée chez nos malades, puisque le pourcentage des yeux dont l'AV était inférieur à 1/10 est passé de 75,75% avant traitement à 19,7% après traitement, alors que le pourcentage des yeux dont l'AV est supérieur à 1/10 est passé de 62,12% à 80,3%. Toutefois, l'aggravation de l'acuité visuelle était notée dans 10,6% des yeux. Il faut noter que les patients dont l'AV après traitement est <1/10 (19,6%) sont majoritairement de sexe masculin (76,9%). 8 patients étaient perdus de vue après la première hospitalisation (24,3% des cas).

Le nombre de poussées par an variait de 0 à 3/an. Les nouvelles poussées d'uvéites et l'extension des lésions oculaires étaient favorisées par la dégression de la corticothérapie orale (corticodépendance), la mauvaise observance du traitement en raison du manque des moyens et du coût élevé du traitement. Les causes infectieuses (foyers infectieux dentaires et ORL, infection urinaire) étaient notées dans 8 cas. Cependant, les causes des poussées ou des rechutes ne pouvaient être expliqué (bilan infectieux négatif, 1ère poussée de la maladie) dans 16 cas. Les complications ophtalmologiques retrouvées chez nos malades soit d'emblée au cours de l'hospitalisation, soit au cours de l'évolution sont présentés dans le Tableau 3.

**Tableau 3:** Comparaison des différentes manifestations ophtalmologiques en fonction du sexe dans notre série

	Totalité des cas 33		Hommes 23		Femmes 10	
	Nombre d'yeux 59	%	Nombre d'Yeux 46	%	Nombre d'yeux 13	%
**Uvéite**	45	77, 3	38	82,6	7	53,8
Antérieure	5	9,1	4	8,7	1	7,7
Intermédiaire	2	3,3	1	2,2	1	7,7
Postérieure	12	20,3	12	26,1	0	0
Panuvéite	33	56	25	54,3	8	61,5
**Neuropathie optique**	28	47,4	20	43,5	8	61,5
**Atteinte vasculaire rétinienne**						
Vascularite rétinienne	32	54,2	29	63	3	23
Occlusion vasculaire	4	6,7	4	8,7	0	0
**Maculopathie**	30	50,8	27	58,6	3	23
Œdème maculaire	22	37,2	21	45,6	1	7,7
Trous et pseudo trous	4	6,7	2	4,3	2	15,3
Membrane épimaculaire	4	6,7	4	8,7	0	0

La comparaison des différentes atteintes oculaires avec cécité et sans cécité, nous a permis de conclure à des facteurs de risque qui sont: le sexe masculin, les antécédents familiaux de maladie de Behçet, la localisation oculaire initiale bilatérale du Behçet, une acuité visuelle initiale inférieure à 1/10, l'atteinte du segment postérieur, la vascularite rétinienne et ses complications ophtalmologiques notamment l'occlusion vasculaire rétinienne, l'ischémie et la néovascularisation, la cataracte et le glaucome. Nous avons essayé de déterminer les particularités épidémiologiques des manifestations ophtalmologiques de la maladie de Behçet, et de comparer ces données entre les deux sexes. Les Tableau 2 et Tableau 4 représentent les différentes manifestations et complications ophtalmologiques de la maladie en fonction du sexe des malades de notre série.

**Tableau 4:** Comparaison des différentes complications ophtalmologiques en fonction du sexe dans notre série

	Totalité des 33 cas		Hommes 23		Femmes 10	
Complications ophtalmologiques	Nombre n= 59 yeux	Pourcentage%	Nombre n= 46 yeux	Pourcentage%	Nombre n= 13 yeux	Pourcentage%
Cataracte	18	30,5	17	37	1	7,7
Synéchie iridocristalliniennes	11	18,6	6	26	5	38,4
Secclusion pupillaire	6	10,1	5	21,6	1	7,7
Hypertonie oculaire	10	16,9	6	26	4	30,7
Glaucome	8	13,5	5	21,6	3	23
Rubéose irienne	1	1,7	1	2,1	0	0
Ischémie rétinienne	5	8,4	5	21,6	0	0
Trou maculaire	4	6,7	2	4,3	2	15,3
Dégénérescence maculaire	19	32,2	17	37	2	15,3
Pâleur papillaire	4	6,7	4	8,7	0	0
Néovaisseaux	3	5	3	6,5	0	0
Hémorragie de vitré	2	3,3	2	4,3	0	0
Décollement de rétine	8	13,5	6	26	2	15,3
Cécité	13	22	11	24	2	15,3

## Discussion

La fréquence de l'atteinte oculaire au cours de la maladie de Behçet est diversement appréciée selon les auteurs et selon le mode de recrutement des patients: elle va de 29% à 100% [4–10] (Tableau 5). La prédominance masculine de notre série est retrouvée par les autres séries de la littérature, avec une nette prédominace des formes symptomatiques et graves chez l'homme (sex ratio de 7/10) [8, 11, 12].

**Tableau 5:** Fréquence de l'atteinte oculaire selon les séries

Série	Nombre de cas	% d'atteinte oculaire
Hamzaoui et al [2]	519	32%
Ouazzani et al [5]	123	100%
El Belhadji et al [6]	520	80%
Filali-Ansary et al [7]	162	51%
Cochereau-Massin et al [9]	41	68%
Janati et al [10]	113	44,2%
Notre série	33	100%

l'âge de début de la maladie de nos patients est comparable aux autres séries. En effet, quelle que soit la population étudiée, la maladie de Behçet s'installe généralement à la troisième décade de la vie [7, 13, 14]. Chez tous ces patients, le diagnostic de la maladie est retenu avant l'âge de 16 ans: la fréquence des formes juvéniles varie de 7% à 44% [7, 8] (4 patients dans notre série).

La fréquence des formes familiales varient entre 2 et 18% selon les populations. Elles semblent être plus graves que les formes sporadiques et sont fortement associées à l'antigène HLA B51. Dans notre série, 2 cas familiaux ont été notés. Il s'agissait dans les deux cas d'une famille avec deux frères.

L'atteinte oculaire survient en moyenne 2,3 ans après l'apparition des signes cutanés. Dans notre série, et pour 72% des malades, l'atteinte oculaire est survenue 32 mois en moyenne après l'apparition des signes cutanés. Toutefois, l'atteinte oculaire peut être inaugurale de la maladie dans 8 à 71% des cas.

Au début, l'atteinte oculaire est souvent unilatérale dans 50 à 60% des cas. Cependant, la bilatéralisation des lésions survient à plus ou moins longue échéance. Dans notre série, l'atteinte oculaire était d'emblée bilatérale dans 79% des cas, ce taux particulièrement élevé dans notre série pourrait être expliqué par la consultation tardive des malades (délai moyen de consultation de 135 jours, avec des extrêmes allant jusqu’à 3 ans), et le recours fréquent à l'automédication pour certains.

Le signe fonctionnel ophtalmologique le plus fréquemment retrouvé et qui a motivé la consultation de nos malades était la baisse de l'acuité visuelle (BAV) (77,3%). Qu'elle soit brutale ou progressive, la BAV peut s'expliquer par un retard de consultation (135 jours en moyenne) et par le recours à l'automédication (corticoïdes collyres) devant des signes oculaires jugés banaux par nos patients: un ‘il rouge douloureux, un brouillard visuel, une sécheresse oculaire ou des myodésopsies, alors que les lésions oculaires sont sévères dans le cadre d'une maladie évolutive. Elle peut être attribuée entre autres à la maculopathie, déjà installée mais sous estimée vu les lésions oculaires associées qui rendent difficile l'exploration du fond doeil (cataracte, hyalite, synéchies irido-cristaliniennes). Ceci doit nous amener à sensibiliser les malades porteurs de maladie de Behçet, à les éduquer pour pouvoir assurer un suivi ophtalmologique régulier.

L'AV initiale <1/10 a été estimée à 37,9% dans notre série, chose qui rejoint notre hypothèse de retard diagnostique. L'atteinte uvéale est la plus fréquente des manifestations ophtalmologiques, retrouvé chez 45 yeux dans notre étude, soit dans 77,3%, elle représente environ 11,4% de toutes les uvéites hospitalisées au service d'ophtalmologie du CHU Hassan II durant la même période. Cette atteinte uvéale représente 1,7 à 15% des étiologies des uvéites [14–18].

L'atteinte du segment postérieur sous forme d'uvéite postérieure ou de panuvéite est l'aspect le plus fréquent dans la maladie de Behçet rapporté par toutes les séries de littérature, et également dans notre étude. L'uvéite antérieure est plus fréquente chez les enfants avant l'âge de dix ans alors que les panuvéites sont plus fréquentes après dix ans (Tableau 6).

**Tableau 6:** Fréquence de l'atteinte uvéale selon différentes séries

Série	Spécialité	N de cas	Atteinte oculaire (%)	Uvéite Antérieure	Hypopion	Uvéite postérieure	Panuvéite
Daghfous et al, Tunisie, (1980) [20]	ophtalmologie	42	85		21,9	10	70,7
Cochereau-Massin et al, France, (1992), [9]	ophtalmologie	41	68	53	6	-	-
Tugal-Tutkun et al, 2004, Turquie, [70]	ophtalmologie	880	100	11	12	28,2	60,2
Ouazzani et al, Maroc, 1995 [5]	ophtalmologie	123	100	-	-	-	72
Filali Ansary et al, Maroc, (1999) [7]	Médecine interne	162	50,6	20,3	-	14,8	
Belhadji et al, Maroc, (1997) [6]	ophtalmologie	520	80	30	6,3	45	37
Janati et a, Maroc, l (2004) [83]	dermatologie	113	44,2	20	8	42	24
B'chir Hamzaoui S et al. Tunisie. (2006) [2]	Médecine interne	519	32,2	12	-	12	45,5
Notre série 2007-2009	ophtalmologie	33	100	-	9,1	18,2	56,1

L'hypopion, souvent associé à un tyndall de chambre antérieure, est le signe le plus évocateur, présent dans 6 à 12% des cas [9, 10, 12]. Mais il n'est ni pathognomonique ni constant au cours de la maladie de Behçet, sa fréquence dans notre série est de 9,1%.

La rétinite, également décrite comme des exsudats rétiniens profonds situés soit en périphérie rétinienne, soit dans la région maculaire, est le deuxième signe retrouvé dans l'uvéite du Behçet (5,8 à 15%). Les foyers de choriorétinite, ont été notés dans 13,6% des yeux dans notre série. La maladie de Behçet domine les étiologies des vascularites rétiniennes 37 à 53,9% [19]. Les lésions vasculaires rétiniennes sont très fréquentes au cours de la maladie de Behçet. Leurs fréquences varient selon les auteurs de 11% à 100%. Elles sont dominées par la vascularite rétinienne essentiellement la périphlébite aussi bien au pôle postérieur qu’à la périphérie rétinienne, présente dans 19,5 à 53% des cas (54,2% dans notre série.

L'atteinte artérielle, plus rare, peut s'associer d'emblée aux lésions veineuses. Les thromboses vasculaires rétiniennes plus fréquemment veineuses qu'artérielles sont plus rares 2 à 10% [9]. L'atteinte inflammatoire du nerf optique est rapportée dans les différentes séries de littérature 7 à 43% [20–22]. Elle peut même être révélatrice de la maladie. Dans notre série, elle a été notée dans 47,4%. Toutefois, la fréquence de la neuropathie optique peut être sous-estimée lorsque le fond doeil est inaccessible du fait d'une uvéite antérieure ou d'une hyalite dense.

La maculopathie au cours de la maladie de Behçet est fréquente, observée dans 16 à 50% selon les auteurs [10, 23], elle est de 50,8% dans notre série. L'atteinte maculaire serait constante au cours de l'atteinte oculaire de la maladie et sa fréquence reste sous-estimée du fait des lésions oculaires associés qui gênent la visibilité du fond doeil. Les manifestations oculaires mineures (conjonctivite, kératite, épisclérite et sclérite, myosite) n'ont pas été retrouvées dans notre série. Elles occupent une fréquence variable de 2,4% à 7,8% selon les travaux [24–26].Dans notre série, l'atteinte cutanéo-muqueuse prédominait les manifestations extra-ophtalmologiques, chose qui rejoint les autres séries [7, 14].

Ceci s'explique par le fait que l'atteinte cutanéo-muqueuse est un critère majeur de diagnostic à rechercher systématiquement devant toute suspicion de maladie de Behçet. L'atteinte vasculaire ayant touché 6,1% des malades, et articulaire (27,3%) ne sembleraient pas influencer la sévérité de l'atteinte oculaire, mais plutôt régressives après un traitement à base d'anticoagulants et une surveillance clinique. Pour ce qui est de l'atteinte neurologique, certains auteurs [8] excluent les céphalées de l'atteinte neurologique, ce qui explique les différences notées. Dans notre série, les céphalées étaient présentes dans 12,1% des cas. Les autres manifestations (digestives, rénales, pulmonaires) n'ont pas été retrouvées dans notre série, vu le faible échantillon de la série, et la prise en charge multidisciplinaire des malades.

Le traitement instauré chez nos malades était avant tout celui de la maladie de Behçet. La corticothérapie (en collyres, par voie orale, par voie injectable et en injections péri-oculaires) était administrée pour juguler l'inflammation dans 97% des cas. Traitement auquel les immunosuppresseurs ont été adjoints, en fonction de la gravité de l'atteinte, la répétition des poussées et la bilatéralité des lésions. La prescription d'immunosuppresseurs à visée de sevrage cortisonique et de récupération fonctionnelle s'avère donc justifiable, mais leur latence d'action impose leur association avec la corticothérapie. Pour certains, l'emploi rapide d'un immunosuppresseur améliore le pronostic à long terme. Le choix des immunosuppresseurs dans notre contexte se base sur l'efficacité du traitement, son accessibilité, la possibilité d'assurer un suivi régulier aux malades pour lesquels il va être prescrit et son coût.

L'indication d'un traitement immunosuppresseur a été posée chez 72,7% de nos malades en cas de vascularite en collaboration avec le service de Médecine Interne. Notre conduite à tenir thérapeutique est similaire à ce qui est décrit dans la littérature, excepté l'usage de l'infliximab pour les uvéites réfractaires, vu le coût de ces molécules, la non disponibilité en milieu intra hospitalier et le bas niveau socio-économiques de nos patients.

Les complications oculaires de la maladie de Behçet sont secondaires aux poussées inflammatoires, à l'évolutivité des lésions et au traitement anti-inflammatoire. Elles sont dominées par: la cataracte 9,75 à 38,5% [7, 12], le glaucome (inflammatoire, cortisonique, ou néo vasculaire: 2,4 à 13% [7, 9] et le décollement de rétine rapporté dans 1,3 à 12% [12].

En l'absence de traitement et de mauvaise observance, le pronostic oculaire de la maladie de Behçet est très mauvais. La cécité s'installe dans 13 à 32% [8, 9]. Le pourcentage élevé de cécité de 22% dans notre série s'explique par le fait que nos malades présentaient des lésions initiales avancées, et que l'aggravation des lésions oculaires sous traitement (immunosuppresseur en particulier) notée dans 6,1% des yeux est mise sous le compte du génie évolutif propre de la maladie. Une étude récente réalisée au « Massachusetts Eye Research and Surgery Institution » portant sur 168 patients atteints de maladie de Behçet colligés au sein de cinq centres d'ophtalmologie, afin de déterminer les facteurs de risque de cécité de la maladie, l'incidence des complications oculaires et de la perte de l'acuité visuelle, a conclus à ce que l'inflammation active de la chambre antérieure SUPOREQUAL 1+, la présence de cellules dans le vitré SUPOREQUAL 2+, un Tyndall vitréen SUPOREQUAL 1+, la présence de synéchies postérieures, d'hypotonie, une PIO élevée étaient associés significativement à une augmentation du risque de la perte de l'acuité visuelle de 20/50 ou plus [25].

Les facteurs de risque retrouvés dans notre série (sexe masculin, les antécédents familiaux de maladie de Behçet, la consultation tardive, AV <1/10, localisation bilatérale du Behçet oculaire, les complications oculaires en particulier glaucome et cataracte) ne sont pas statistiquement significatifs vue le faible échantillonnage.

La sévérité et le nombre des poussées d'uvéites en particulier l'inflammation du segment postérieur détermine l'importance des changements structuraux permanents et le taux résultant de cécité [12]. Cependant, le typage HLA B51 n'est prédictif de la survenue d'une atteinte oculaire, et de la sévérité du pronostic oculaire [9].

L'influence du sexe sur l'expression clinique et la sévérité de la maladie de Behçet et en particulier de l'atteinte oculaire soulèvent le rôle aggravant des androgènes [26]. Dans notre série, l'atteinte oculaire prédominait chez les patients de sexe masculin (69,7%). L'uvéite (82,6%), la vascularite rétinienne (63%) et l'occlusion vasculaire rétinienne (8,7%), loedème maculaire (54,3%) prédominaient chez les patients de sexe masculin. Les complications les plus graves ont été notées chez les hommes de notre série. En plus, 76,9% des patients qui ont gardé une acuité visuelle finale <1/10 était de sexe masculin.

## Conclusion

Les manifestations ophtalmologiques au cours de la maladie de Behçet ont une valeur considérable, tant sur le plan diagnostique que pronostique. La sémiologique clinique est variable et regroupe différentes atteintes uvéales et vasculaires rétiniennes qui sont le plus souvent intriqués. Notre étude a permis de décrire les aspects cliniques de l'atteinte oculaire de la maladie de Behçet au service d'Ophtalmologie du CHU Hassan II de Fès. Les résultats de notre série sont globalement comparables à celles publiées par les différentes séries rapportant la maladie. Il est important d'insister sur l'urgence d'un traitement précoce et énergique, seul en mesure de faire disparaître les symptômes inflammatoires, d'empêcher les récidives et de permettre une amélioration de l'état visuel. Une surveillance ophtalmologique de la maladie est particulièrement importante afin de rechercher précocement les lésions du segment postérieur qui sont de loin les manifestations les plus graves. Le pronostic oculaire de la maladie de Behçet peut être amélioré par une prise en charge précoce et un suivi clinique et angiographique rigoureux. La collaboration étroite entre ophtalmologistes et internistes est particulièrement importante afin de préserver l'avenir visuel des patients. Des études supplémentaires à plus grande échelle sont nécessaires afin de préciser les particularités de l'atteinte oculaire de cette maladie possiblement cécitante.
